# Automatic Pancreatic Ductal Adenocarcinoma Detection in Whole Slide Images Using Deep Convolutional Neural Networks

**DOI:** 10.3389/fonc.2021.665929

**Published:** 2021-06-25

**Authors:** Hao Fu, Weiming Mi, Boju Pan, Yucheng Guo, Junjie Li, Rongyan Xu, Jie Zheng, Chunli Zou, Tao Zhang, Zhiyong Liang, Junzhong Zou, Hao Zou

**Affiliations:** ^1^ Department of Automation, School of Information Science and Engineering, East China University of Science and Technology, Shanghai, China; ^2^ Department of Automation, School of Information Science and Technology, Tsinghua University, Beijing, China; ^3^ Molecular Pathology Research Center, Department of Pathology, Peking Union Medical College Hospital (PUMCH), Peking Union Medical College and Chinese Academy of Medical Sciences, Beijing, China; ^4^ Yihai Center, Tsimage Medical Technology, Shenzhen, China; ^5^ Center for Intelligent Medical Imaging & Health, Research Institute of Tsinghua University in Shenzhen, Shenzhen, China; ^6^ Shanghai Chenshan Plant Science Research Center, Chinese Academy of Sciences, Shanghai, China

**Keywords:** pancreatic ductal adenocarcinoma (PDAC), histology, deep learning, convolutional neural network, whole-slide image analysis

## Abstract

Pancreatic ductal adenocarcinoma (PDAC) is one of the deadliest cancer types worldwide, with the lowest 5-year survival rate among all kinds of cancers. Histopathology image analysis is considered a gold standard for PDAC detection and diagnosis. However, the manual diagnosis used in current clinical practice is a tedious and time-consuming task and diagnosis concordance can be low. With the development of digital imaging and machine learning, several scholars have proposed PDAC analysis approaches based on feature extraction methods that rely on field knowledge. However, feature-based classification methods are applicable only to a specific problem and lack versatility, so that the deep-learning method is becoming a vital alternative to feature extraction. This paper proposes the first deep convolutional neural network architecture for classifying and segmenting pancreatic histopathological images on a relatively large WSI dataset. Our automatic patch-level approach achieved 95.3% classification accuracy and the WSI-level approach achieved 100%. Additionally, we visualized the classification and segmentation outcomes of histopathological images to determine which areas of an image are more important for PDAC identification. Experimental results demonstrate that our proposed model can effectively diagnose PDAC using histopathological images, which illustrates the potential of this practical application.

## Introduction

Pancreatic ductal adenocarcinoma (PDAC) is a highly malignant tumor of the digestive system with few symptoms until the cancer is advanced (1). It ranks as the seventh leading cause of cancer death in both sexes combined (2), and most patients die within 2 years of the initial diagnosis (3, 4). Due to the lack of early diagnosis and effective treatment, the prognosis of patients with PDAC is extremely poor (5–7). The latest cancer survival data show that the overall 5-year survival rate of PDAC is 9% (2), which is the lowest among all kinds of cancers. In recent years, because of the changes in people’s dietary habits and lifestyles associated with rapid economic growth, the incidence of PDAC has dramatically increased year by year (8). However, the diagnosis of PDAC is still a challenge for pathologists, especially for the well-differentiated adenocarcinoma, whose clinical histological patterns are similar to those of chronic pancreatitis (9). Many studies have focused on the development of diagnostic biomarkers for distinguishing between pancreatitis and PDAC (10, 11). Unfortunately, the effectiveness of available diagnostic biomarkers is limited. Thus, we need a novel method that could provide an adjuvant diagnosis of PDAC efficiently and accurately to allow timely treatment.

With recent developments, digital medical imaging has played an indispensable role in PDAC diagnosis and treatment planning. Multi-detector computed tomography (CT), magnetic resonance imaging (MRI), and endoscopic ultrasound are the recommended initial imaging techniques for making a timely diagnosis of PDAC (12). Although, the gold standard for clinical medical diagnosis is histopathological image evaluation by pathologists (13), this is a manual and time-consuming procedure with several drawbacks. The principal limitation is the severe shortage of senior pathologists all over the world (14), since the accuracy of diagnosis depends on the professional knowledge and clinical diagnostic experience of the pathologist, which can lead to low diagnosis concordance (15). Moreover, pathologists now spend considerable time on benign biopsies that represent approximately 80% of all cases (16). Thus, there is an urgent need to develop automatic adjuvant diagnostic methods that could distinguish between benign and cancerous tissues in PDAC.

In the past three decades, much progress has been made in data storage and computation capacity. Graphics processing units have undergone rapid development. They offer a powerful parallel computing capability, especially for studies with many samples (17). Moreover, it is possible to digitize glass slides, such as whole-slide imaging (WSI) (18). These advances have led to the development of automatic diagnosis methods using medical image analysis (19). Currently, automatic diagnosis approaches have received widespread attention and made significant progress in detecting breast cancer (20–23), for colonography (24), and in assessing lung nodules (25). Some researchers have even proposed automatic diagnostic methods for COVID-19 (26). Automatic systems can assist in diagnosis, thus reducing the workload, increasing diagnostic efficiency, and preventing missing inspections. This would allow pathologists to pay more attention to providing oversight and quality functions rather than making primary diagnoses (27).

However, relatively little research has been done on the automatic analysis of pancreatic histopathological images. A significant reason may be the lack of publicly available datasets with pancreatic histopathological images, especially datasets large enough for training convolutional neural networks (CNNs). Nevertheless, some scholars have studied the automatic diagnosis of pancreatic cancer from CT and MRI images. Chen et al. (28) proposed a three-stage modified form of Faster R-CNN for recognizing and classifying cystic pancreatic neoplasms using MRI images of the abdomen, which yielded an accuracy of 92.3% in patient-level classification. Recently, Xuan et al. (29) presented a hierarchical CNN for pancreatic tumor detection from MRI images. However, unlike an analysis of WSIs, detecting PDAC with CT or MRI images requires an initial segmentation of the pancreas, which increases the computing resources needed and decreases efficiency. Moreover, some studies have attempted to automatically detect pancreatic cancers with WSIs based on feature extraction methods. Change et al. (30) used paired pancreatic histopathological and immunofluorescence images to classify nuclei. Song et al. (31) proposed a model for automatically grading pancreatic adenocarcinoma using morphological features. They segmented a PDAC tissue image into the lumen, epithelial nuclei, and non-epithelial nuclei, and then extracted several morphological features from the epithelial cells and segmented lumen parts, achieving an accuracy of 94.38% in binary classification. Langer et al. (32) developed a method for detecting early pancreatic lesions in mice, realizing a 93% success rate with the test dataset. They incorporated a feature analysis of ducts, nuclei, and tumor stroma when training the model. Le et al. (33) used a noisy label classification method to predict regions of pancreatic adenocarcinoma in WSIs. However, feature-based classification methods are applicable only to a specific problem and lack versatility. Moreover, there are many difficulties in designing and extracting relevant pathological characteristics. Thus, deep learning is becoming a vital alternative to feature extraction.

In this paper, we propose a novel automatic method for detecting PDAC in WSIs based on CNNs. To the best of our knowledge, this is the first CNN architecture for PDAC detection trained on a relatively large WSI dataset, whose purpose was to determine the potential of machine learning methods on automatic PDAC diagnosis in WSIs. The remainder of this paper is organized as follows. *Materials and Methodology* introduces the dataset and our deep-learning-based PDAC diagnostic framework. Then, the methodology applied in this study is described in detail. After that, our experiments and results are provided in *Experiments and Results*. Finally, the discussion is presented in the section *Discussion*.

## Materials and Methodology

### Dataset and Annotation

**Figure 1:**
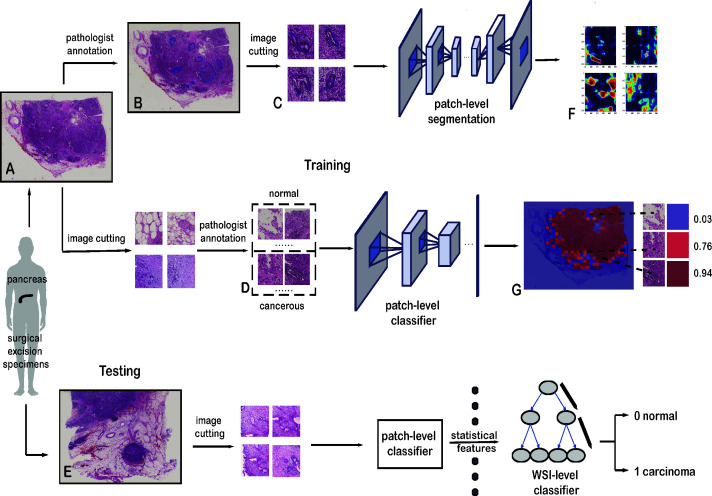
Framework of the deep-learning approach. **(A)** Training data with raw WSIs. **(B)** Annotated WSIs. **(C)** Patches for training the patch-level segmentation. Each patch has a region with carcinoma. **(D)** Two classes of patches for training the patch-level classifier. **(E)** Testing data with raw WSIs. **(F)** Heatmap, as the output of the patch-level segmentation. **(G)** Malignant probability heatmap.

In this study, 60 normal and 171 cancerous pancreas tissue samples were selected as our dataset. All the pancreatic image samples were collected and their use authorized by Peking Union Medical College Hospital (PUMCH). Each specimen was stained by hematoxylin and eosin and saved as an uncompressed high-resolution WSI (**Figure 1A**). These were labeled and confirmed by a senior pathologist from PUMCH. The study protocol was approved by the ethics review board of PUMCH.

All the slides in our dataset were digitized with a scanner (KF-pro-400, Ningbo, China) under the same acquisition conditions with a magnification of 40× (0.2 µm/pixel). WSIs are multi-gigabyte images with typical resolutions of 100,000 × 100,000 pixels, though each WSI has a different size. Increasing the size of the input images would have increased the number of parameters to be estimated, as well as the required computational power and memory (34). In this study, small patches were sampled from WSIs at high magnification. These patches were used to train the patch-level classification and segmentation models:

1. The patch-level classification dataset has two sampling sets: a positive set and a negative set. The positive set has patches within cancerous cells and lesions, whereas the negative set has patches with normal cells and tissue. These two sets were sampled from 60 normal and 30 cancerous WSIs. They were cropped to 1,024 × 1,024 pixels. Pathologists then categorized these patches into the positive or negative set, shown in **Figure 1D**.
2. For the patch-level segmentation, the dataset comprised six WSIs. Each whole PDAC area was annotated in blue by a pathologist (**Figure 1B** and **Supplementary Figure 2**). We then found the minimum bounding box of each annotated region. These rectangles were cut into small patches of 1,024 × 1,024 pixels. To ensure each patch included an area sampled from the annotated regions, we removed some patches that were only sampled outside the annotated regions. Some patches are shown in **Figure 1C**.

Artificial intelligence for classifying pathology images is heavily dependent on the scale of the training dataset. To avoid overfitting and generalizing, a large amount of data is required for training a complex network. However, there are several barriers to obtaining digital pathology images from a clinical laboratory (35). Since we had insufficient raw data, we augmented the data by rotating patches through various angles as well as flipping and reflecting them. The label for each patch generated was inherited from its parent. To acquire balanced data for training, validation, and testing, the scale of augmentation in the two classes depended on the number of patches. The distribution of patches we used for training the patch-level classification model is shown in **Table 1**.

**Table 1:** Distribution of patches extracted from the raw WSIs for training the patch-level classifier.

Class	Testing	Training	Validation	Total
Normal	4,988	39,900	4,988	49,876
Carcinoma	4,960	39,688	4,964	49,612
Total	9,948	79,588	9,952	99,488

### Model Architecture

In our study, a novel deep-learning framework was designed to classify pancreatic histopathological images. Overview of the proposed study consisted of two parts: classification and segmentation of PDAC detection in WSIs. Meanwhile, classification and segmentation are two separate tasks. The segmentation method can be seen as an ancillary or control study.

The classification method has a two-step framework based on diverse recognition objects, and the training process can be divided into two stages, patch level and WSI level. For the patch-level classification, we employed a CNN model to extract the hidden features from the training set and then applied the model to the test data. For each WSI, we predicted the relevant patches with the trained patch-level classifier. And these predicted patches were combined into a malignant probability heatmap. Next, we mapped the cancer probability of each patch into colors between dark blue and crimson (**Figure 1G**). For the WSI-level classification, 36 statistical features of these malignant probability heatmaps were harnessed to train a Light Gradient Boosting Machine (LightGBM) (36) model for the identification and diagnosis of PDAC.

For the patch-level segmentation, a fully convolutional network, U-Net (37), was selected to predict and locate the cancer regions. A detailed framework of the segmentation method is shown in **Supplementary Figure 3**. Additionally, we visualized the outcomes from the patch-level classification and patch-level segmentation models. Visualizing the output of a CNN layer can show what the model has learned, which is a vital function of the deep-learning model. Finally, we validated the performance of our model with an independent dataset. A summary of the architecture applied in our study is shown in **Figure 1**.

### Patch-Level Classification

CNNs are feed-forward neural networks that are widely applied for visual pattern recognition. In this study, we chose a well-known CNN framework, Google’s Inception V3 (38), as the patch-level classifier. Inception V3 has been extensively adopted for different kinds of digital histopathological image analysis, such as for bladder (39), breast (40), and liver (41). In contrast, state-of-the-art CNNs have not been widely used for the classification of pancreatic histopathological images.

In our research, besides the basic Inception V3 model, we exploited a global average pooling layer, a full connection layer of 1,024 neurons, and a softmax layer to obtain the final classification results. The Inception V3 model, which has about 25 million parameters, was trained on the training data. Then the trained model was applied to classify pancreatic histopathological images in the test data. The network weights were initialized randomly, and the learning rate of the gradient-descent back-propagation was 0.001. During training, Adam was selected as the optimizer, as it has been widely applied in training Inception V3 models due to its fast convergence. Additionally, we chose the categorical cross-entropy as the loss function. The classifier was trained for 100 epochs, and the batch size of each epoch was eight. To optimize the performance of our model, we took the set of parameters with the highest accuracy in the validation data as the final parameters.

### WSI-Level Classification

**Figure 2:**
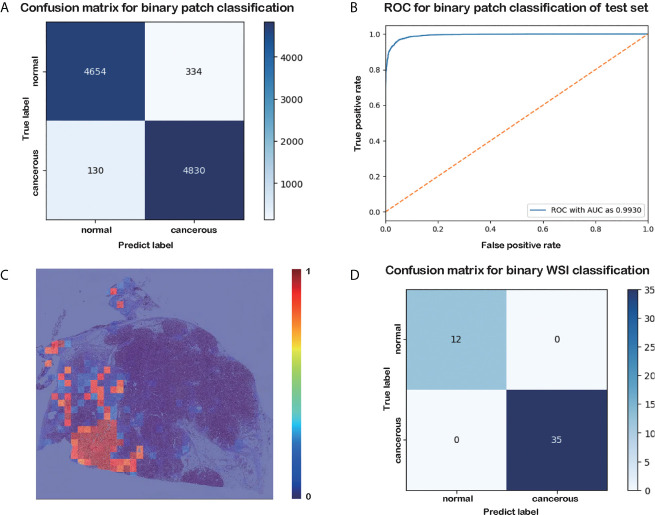
Results of the patch-level classification for the test data. **(A)** Confusion matrix for binary patch classification. **(B)** ROC. **(C)** Heatmap of cancer probability generated by our trained classifier. **(D)** Confusion matrix for binary WSI classification.

For each WSI, a cancer probability heatmap was generated based on the results of the patch-level classification. The probability that there is pancreatic cancer in a patch was between 0 (predicted to be normal tissue or the background) and 1 (predicted to be a cancerous region). Next, we mapped the cancer probability into colors in a continuous range between dark blue and crimson. To clearly show the cancerous tissue predicted by our model, we overlaid the original WSI with the cancer probability heatmap, as shown in **Figure 2C**.

After obtaining the cancer probability heatmaps, we post-processed the data before training the WSI-level classifier. We extracted 36 statistical features from each cancer probability heatmap, such as the mean, variance, and sum, as listed in **Table 2**. More detailed descriptions of these features are given in **Supplementary Table 1**, and the importance of these features is given in **Supplementary Figure 1**. Then, we used the LightGBM model for the WSI-level classification (42). LightGBM is a gradient-boosting framework based on decision trees. It is an efficient model with low memory usage, which is required for automatic histopathological image analysis in clinical practice. Limited by memory and computation time, our model is not an end-to-end approach for WSI-level classification. All the training WSIs were the same as those chosen as the training data for the patch-level classification.

**Table 2:** The 36 features extracted from a heatmap of malignant probabilities at the WSI-level.

Feature	Description of feature	The number of features
1–9	Mean, variance, standard deviation, median, mode, minimum, maximum, range, sum of normal probabilities	9
10–18	Mean, variance, standard deviation, median, mode, minimum, maximum, range, sum of tumor probabilities	9
19–20	*N_p_* for two classes with *P > 0*.999	2
21–22	*N_p_* for two classes with 0.99 < *P ≤* 0.999	2
23–24	*N_p_* for two classes with 0.95 < *P ≤* 0.99	2
25–26	*N_p_* for two classes with 0.9 < *P ≤* 0.95	2
27–28	*N_p_* for two classes with 0.8 < *P ≤* 0.9	2
29–30	*N_p_* for two classes with 0.7 < *P ≤* 0.8	2
31–32	*N_p_* for two classes with 0.6 < *P ≤* 0.7	2
33–34	*N_p_* for two classes with 0.5 < *P ≤* 0.6	2
35	Numeric label of the category to which the largest value for the mean of *P* belongs	1
36	Numeric label of the category with the most patches	1

### Patch-Level Segmentation

Besides pancreatic histopathological image classification, another vital task in our study is comparing the performance of segmentation and classification in PDAC prediction. This would allow us to choose the most appropriate method for diagnosing a sample in a practical clinical application. In this study, we chose U-Net for the patch-level segmentation. U-Net is an end-to-end architecture comprising a contracting path and a symmetric expanding path. It can capture the context and precisely locate each pixel (43). In this work, to expand the volume of the training dataset and avoid overfitting, patches were subsampled into 256 × 256 pixels with a half overlap ratio before training. Moreover, using smaller patches allows our model to get a better grasp of subtle features. Training the model with half overlap patches made the size of the output data consistent with the input patches, preventing the imperfection of the valid padding method within the U-Net model. The network weights were initialized randomly. We chose the dice coefficient (44) as the loss function.

## Experiments and Results

### Patch-Level and WSI-Level Classification

In this section, we evaluated the performance of our proposed model on the test dataset using the accuracy, precision, recall, and *f*_1_ score. Detailed information on these metrics can be found in (45).

The results of the patch-level classification for the test dataset are listed in **Table 3**. **Figure 2A** shows the confusion matrix for the binary patch classification. The receiver operating characteristic curve (ROC) is shown in **Figure 2B**. Accuracy of 0.9533 was achieved in the patch-level classification, and the recall of cancerous patches was 0.9738, which was higher than the recall of the negative set (0.9330). This result indicates that our model has a high recall for cancerous cases. Although many patches labeled as carcinoma were correctly categorized, many normal patches were also classified as carcinoma, resulting in the lower precision for positive samples. However, this performance is consistent with the requirement for clinical diagnosis, since pathologists must be rigorous and not overlook any patch that could be carcinoma. Consequently, our classification model could be developed into a pre-screening tool for pathologists, indicating suspect areas in pancreatic tissue.

**Table 3:** Performance of patch-level classification.

Class	Accuracy	Precision	Recall	F1-score
Normal	0.9533	0.9728	0.9330	0.9525
Cancerous		0.9353	0.9738	0.9542

As shown in the confusion matrix in **Figure 2D**, the accuracy of the WSI-level classification on the test data was 100%. Our WSI-level test dataset comprised 12 normal and 35 cancerous WSIs, accounting for 20% of the whole WSI dataset. A sample diagram of the test data is shown in **Figure 1E**. Due to the limited memory available, all the WSIs were trained or tested without data augmentation, which means that the dataset was unbalanced. Nonetheless, our proposed method categorized 47 WSIs correctly. This result demonstrates the tremendous potential of our model in a clinical application for analyzing pancreatic histopathological images.

### Patch-Level Segmentation

The distribution of the dataset for patch-level segmentation and the values of the dice coefficient are listed in **Table 4**. We extracted 1,732 patches of 1,024 × 1,024 pixels within the annotated PDAC areas from six WSIs. Next, these patches were subsampled into 256 × 256 pixels with a half overlap ratio before training. This increased the size of our dataset by a factor of 64. During training, random horizontal flips and jitter were harnessed to augment the data further. Finally, the dice coefficient for the validation data and test data was 0.7602 and 0.8465, respectively.

**Table 4:** Performance of patch-level segmentation.

	Training	Validation	Test	Total
	1,385	184	163	1732
Average dice	–	0.7602	0.8465	–

**Figure 3:**
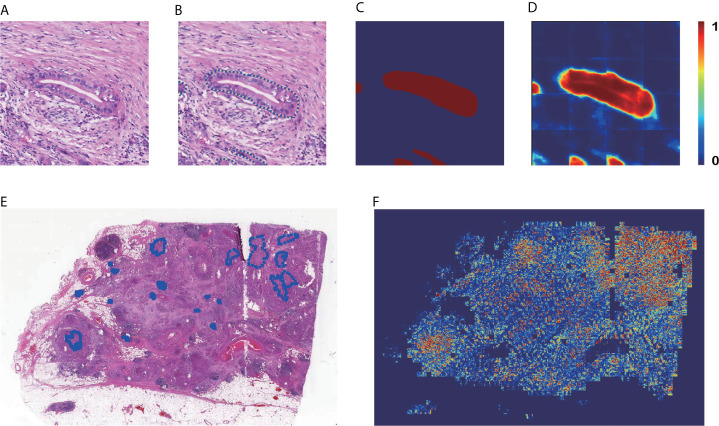
Results of patch-level segmentation. **(A)** A sample raw patch. **(B)** Annotated patch. **(C)** Mask generated by the annotation. **(D)** Heatmap of the sample patch predicted by our method. **(E)** A sample WSI with annotation. **(F)** Heatmap of the sample WSI comprising the heatmaps predicted for each patch.

**Figures 3A–D** are the input and output of the patch-level segmentation, where **Figure 3A** shows an original sample patch extracted from a WSI, and **Figure 3B** shows PDAC areas annotated with blue circles by a pathologist. Then we transformed the annotated information into a mask, as illustrated in **Figure 3C**. The region with carcinoma is crimson, whereas the background and normal tissue are dark blue. In this study, the patch-level segmentation was trained with original patches and masks. The result is a cancer probability matrix of size 256 × 256, from which we created a heatmap, as shown in **Figure 3D**. The cancer probability is represented with a continuous range of colors between dark blue and crimson. **Figure 3D** indicates that our segmentation model has a high sensitivity for PDAC since most PDAC regions are predicted correctly. However, some PDAC regions are predicted to be normal tissue, indicating that the segmentation for these was inaccurate.

**Figures 3E, F** are examples of WSI-level segmentation. As well as the cancer probability heatmap from the WSI-level classification, the WSI-level segmentation heatmap includes the patch-level segmentation results, as shown in **Figure 1F**. Compared with the sample annotated WSI in **Figure 3E**, the predicted WSI segmentation heatmap in **Figure 3F** indicates several PDAC areas correctly, especially at the upper right and lower left. However, it is not precise because the prediction regions always cover a larger area than the true PDAC annotation. This performance is almost consistent with the patch-level classification results. These two deep-learning models give a high sensitivity for cancerous regions., which may cause some normal regions are classified as PDAC, resulting in a high false-positive rate.

### Visualization of Outcomes by Grad-CAM and Heatmap

**Figure 4:**
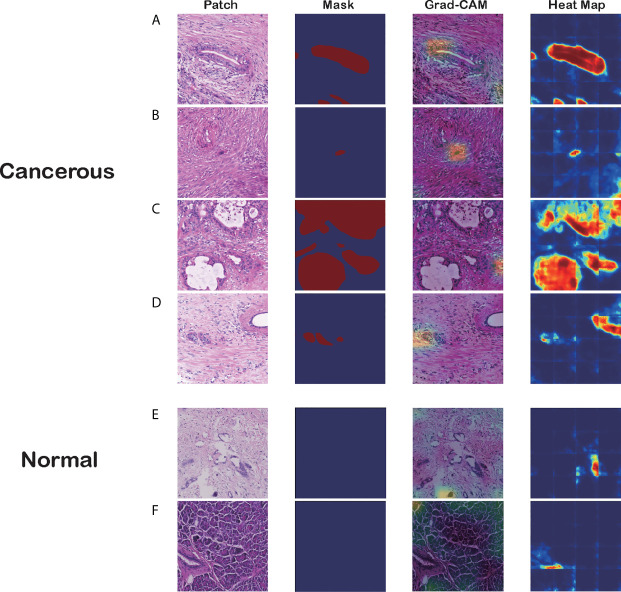
Visualizations of different pancreatic lesions by Grad-CAM and the corresponding heatmaps. **(A–D)** Positive sets with cancerous tissues. **(E, F)** Negative sets with background or normal tissue. The four columns from left to right are patch extracted from WSIs, mask generated from the annotation, Grad-CAM and heatmap presentation for these patches, respectively.

After we obtained the trained patch-level classification and patch-level segmentation models, we visualized the prediction results of these two methods with the same input data, as shown in **Figure 4**. We used Grad-CAM (46), which takes information about the class-specific gradient flowing into the penultimate layer of the CNN model and then generates an attention map demonstrating how intensely the input data activates diverse channels in the layer for the class. This attention map can be regarded as a coarse localization map highlighting the vital areas for CNN model prediction. We compared the Grad-CAM output with the true annotation to investigate whether our model can correctly locate abnormal cells and cancerous tissues.

**Figure 4** has six groups of input images, masks, and visualization results from the two approaches, where groups A to D are positive sets with cancerous tissues, and groups E and F are negative sets with background or normal tissue. Each group comprises four figures: a raw patch sampled from a WSI, a mask generated from the annotation, Grad-CAM output based on the CNN prediction, and a heatmap produced by the segmentation model.

As the figures illustrate, both methods can recognize carcinoma regions effectively. Even if the input patch contains only small portions of carcinoma tissue, our model can detect those positive areas correctly. **Figure 4B** is an example in which the two methods successfully identified a single cancerous nucleus. However, the patch-level segmentation method was more accurate in recognizing tumor tissue. For example, in **Figure 4A**, Grad-CAM finds only the head region of the abnormal nucleus, whereas the segmentation method retrieves the whole nucleus accurately. These results indicate that our algorithm probably recognized adenocarcinoma mainly with nuclear features. Large, irregular, crowded, and dark areas were considered to be tumor cell nuclei by our algorithm. These features are critical for tumor recognition. However, there are also false-positive and false-negative regions, such as regions crowded with nuclei.

Additionally, we discovered that the segmentation model might be making decisions based on the color contrast, whereas the classification model tended to pick out irregular and crowded groups of abnormal cell nuclei. **Figure 4C** demonstrates that the segmentation model identifies most PDAC regions successfully. However, the Grad-CAM has false-positive regions where the cell nuclei distribution is irregular. In **Figure 4D**, the classification model distinguishes the PDAC cell nuclei correctly. In contrast, the heatmap has a false-positive area to the right of the patch with high color contrast. This was verified in the predictions for normal patches.

### Independent Verification

**Figure 5:**
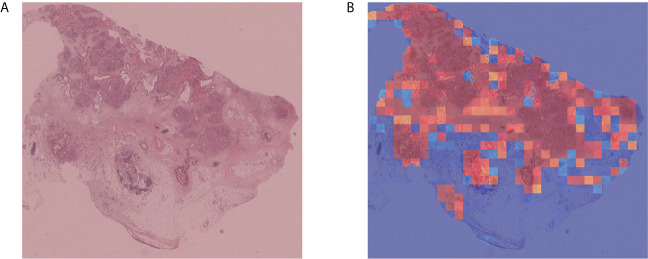
Independent verification results. **(A)** Sample WSI labeled as carcinoma by TCGA. **(B)** Cancer probability heatmap from our WSI classification method.

To verify the portability and robustness of our model, we tested our WSI-level classification method on a public dataset. The Cancer Genome Atlas (TCGA) (47) is a publicly funded project with multi-dimensional information such as genomes, proteomes, and histopathological images of more than 20,000 examples of primary cancer. We downloaded 52 WSIs labeled as carcinoma from TCGA, as their sizes were almost consistent with our WSIs. We applied our trained WSI-level classification model to analyze these WSIs. Our prediction accuracy was 90.38%. Five WSIs were misclassified as negative, whereas all the other WSIs were correctly categorized as carcinoma. Moreover, we plotted cancer probability heatmaps. As shown in **Figure 5**, our approach effectively detected the lesion.

## Discussion

In this study, an automatic method for detecting PDAC using deep CNNs was designed and implemented. Our work has two main highlights (1). This is the first CNN architecture for PDAC detection trained on a relatively large WSI dataset (2). We attempted to understand the decision-making process of the classification and segmentation methods to make the automatic diagnosis more transparent and explainable.

Due to the lack of publicly available datasets with pancreatic histopathological images, relatively little research has been done on the automatic detection of pancreatic adenocarcinoma. Our dataset has 231 WSIs with WSI-level labels. All the relevant features were learned by the CNNs, reducing the time for feature extraction and reducing the requirements for expertise in pathology. Our patch-level approach achieved 95.3% classification accuracy and the WSI-level approach achieved 100% classification accuracy. Moreover, this model has been proven to be generalizable, as it reached 90.38% classification accuracy with an independent verification dataset. Furthermore, our approach shows high sensitivity for carcinoma regions, which is important in assisting clinical diagnosis. Consequently, the heatmap of cancer probability could help pathologists to rapidly notice suspicious regions, which may significantly reduce inspection times and costs and improve the efficiency of the diagnostic process.

Deep learning is often dubbed to be a black box because its decision-making process is not understandable to humans. Thus, we visualized the outcomes of our classification and segmentation methods. From the visualizations of the convolutional layers, we noticed that our algorithm recognized adenocarcinoma mainly from its nuclear features, such as the shape and chromatin characteristics. This information is important for pathologists in making a final diagnosis.

In the future, we will analyze the morphological features of PDAC in light of the existing classification and segmentation model. Our algorithm recognizes adenocarcinoma mainly from the nuclear features. However, the nuclear features of a series of well-differentiated adenocarcinomas are almost indistinguishable from those of pancreatitis. Further, because of the fibrosis in adenocarcinoma stroma, the nuclei of fibroblasts are large and irregular, like tumor cells, so that the algorithm recognized fibroblasts as tumor cells. Additionally, the nuclear features are influenced by how the tissue was fixed and stained, which thus, affected the accuracy. Instead of recognizing adenocarcinomas using magnified nuclear features, it may be better to combine histological patterns and nuclear features, which is common in PDAC diagnosis. This may improve the accuracy of the algorithm. Furthermore, we would like to extract features from the same histopathological images at different magnification levels. If the model captured the structure of adenocarcinoma at high and low magnification, the nucleus of each cell could be recognized more clearly. Our next research direction is to combine multiscale characteristics and statistical features. Several relevant studies are currently ongoing.

Since this study used a two-stage architecture, we recommend that further classification methods could be designed as an end-to-end model if higher computer capacities are available. Nevertheless, our proposed model is an efficient aid for doctors in making quick and accurate identifications and diagnoses of PDAC. Additionally, our findings are potentially applicable for improving the identification and treatment of PDAC and saving a significant amount of pathologists’ time. The intended goal beyond research is to incorporate our proposed method into clinical practice as pre-screening in PDAC diagnosis.

## Data Availability Statement

The raw data supporting the conclusions of this article will be available by the authors without undue reservation.

## Ethics Statement

The studies involving human participants were reviewed and approved by Peking union medical college hospital (PUMCH). Written informed consent for participation was not required for this study in accordance with the national legislation and the institutional requirements.

## Author Contributions

ZL, HZ, JZZ, YG conceived and designed the study. HF, WM, BP, and YG analyzed the data and wrote the manuscript. BP and JL provided the proprietary WSI datasets, their corresponding annotations. JL, RX, JZ, CZ, TZ, JZZ, HZ, and ZL revised the final manuscript and provided direction and guidance throughout the preparation of this study. ZL, HZ, and JZZ supervised the project. All authors contributed to the article and approved the submitted version.

## Funding

This work was supported by the Foundation of Beijing Municipal Science and Technology Commission(Z181100001918004), the National Key Research and Development Program of China (2018YFF0301102 and 2018YFF0301105), and the National Natural Science Foundation of China (Nos.61976091).

## Conflict of Interest

The authors declare that the research was conducted in the absence of any commercial or financial relationships that could be construed as a potential conflict of interest.
