# Supplementary material for: Automatic Pancreatic Ductal Adenocarcinoma Detection in Whole Slide Images Using Deep Convolutional Neural Networks
Source: Front Oncol. 2021 Jun 25;11:665929. doi: 10.3389/fonc.2021.665929 (PMC8267174; doi:10.3389/fonc.2021.665929)
Supplement: Supplementary file 1 [file DataSheet_1.docx]

Supplementary Material

# Supplementary Data

Our dataset contains 231 WSIs, 60 normal and 171 cancerous WSIs were obtained. All the WSIs were collected and authorized by PUMCH. The format of WSIs is *ndpi*. The normal WSIs occupy 77GB of memory space, and the cancerous WSIs occupy 313GB of memory space. All slides are digitalized with KF-pro-400 (Ningbo, China) scanner under the same acquisition condition, with a magnification of 40× (0.2*μm*/pixel). Our independent validation dataset contains 52 WSIs obtained from TCGA. For patch-level segmentation, a senior pathologist of PUMCH annotated 6 WSIs using ASAP.

# Supplementary Figures and Tables

**2.1 Supplementary Tables**

Supplementary Table 1.

**The 36 features extracted from a heatmap of malignant probabilities at the WSI-level.**

| Index | Explanation of feature |  |
| --- | --- | --- |
| Feature 1 | Mean of normal probabilities | |
| Feature 2 | Variance of normal probabilities | |
| Feature 3 | Standard deviation of normal probabilities | |
| Feature 4 | Median of normal probabilities | |
| Feature 5 | Mode of normal probabilities | |
| Feature 6 | Min of normal probabilities | |
| Feature 7 | Max of normal probabilities | |
| Feature 8 | Range of normal probabilities | |
| Feature 9 | Sum of normal probabilities | |
| Feature 10 | Mean of tumor probabilities | |
| Feature 11 | Variance of tumor probabilities | |
| Feature 12 | Standard deviation of tumor probabilities | |
| Feature 13 | Median of tumor probabilities | |
| Feature 14 | Mode of tumor probabilities | |
| Feature 15 | Min of tumor probabilities | |
| Feature 16 | Max of tumor probabilities | |
| Feature 17 | Range of tumor probabilities | |
| Feature 18 | Sum of tumor probabilities | |
| Feature 19 | Proportion of normal patches with predicted values >0.999 | |
| Feature 20 | Proportion of tumor patches with predicted values >0.999 | |
| Feature 21 | Proportion of normal patches with 0.99<predicted values≤0.999 | |
| Feature 22 | Proportion of tumor patches with 0.99<predicted values≤0.999 | |
| Feature 23 | Proportion of normal patches with 0.95<predicted values≤0.99 | |
| Feature 24 | Proportion of normal patches with 0.95<predicted values≤0.99 | |
| Feature 25 | Proportion of normal patches with 0.9<predicted values≤0.95 | |
| Feature 26 | Proportion of normal patches with 0.9<predicted values≤0.95 | |
| Feature 27 | Proportion of normal patches with 0.8<predicted values≤0.9 | |
| Feature 28 | Proportion of normal patches with 0.8<predicted values≤0.9 | |
| Feature 29 | Proportion of normal patches with 0.7<predicted values≤0.8 | |
| Feature 30 | Proportion of normal patches with 0.7<predicted values≤0.8 | |
| Feature 31 | Proportion of normal patches with 0.6<predicted values≤0.7 | |
| Feature 32 | Proportion of normal patches with 0.6<predicted values≤0.7 | |
| Feature 33 | Proportion of normal patches with 0.5<predicted values≤0.6 | |
| Feature 34 | Proportion of normal patches with 0.5<predicted values≤0.6 | |
| Feature 35 | Numeric label of the category to which the largest value in the mean of patch belongs | |
| Feature 36 | Numeric label of the category with the most patches | |

We extracted 36 features for patch-level classification training, the supplementary table1shows the explanation of each feature and its name. **2.2 Supplementary Figures**


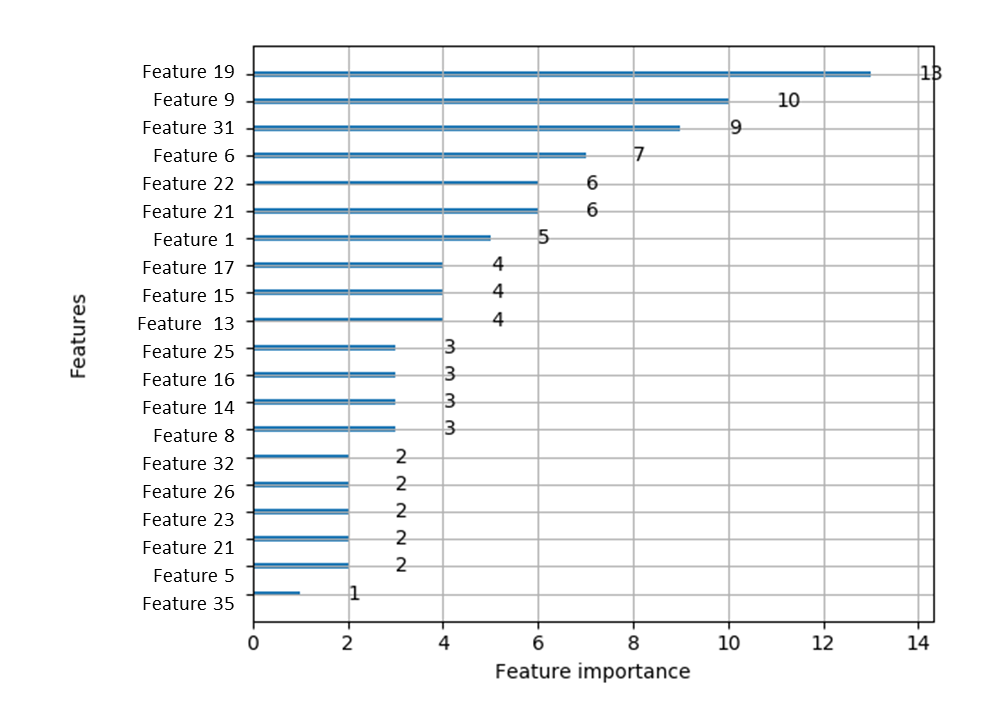


Supplementary Figure 1. **The feature importance of extracted heatmap features**

We extracted 36 features for patch-level classification training, and supplementary figure 1shows the feature importance of the twenty most important features. The sequence number of a feature is corresponding to the feature name listed in Supplementary Table 1.


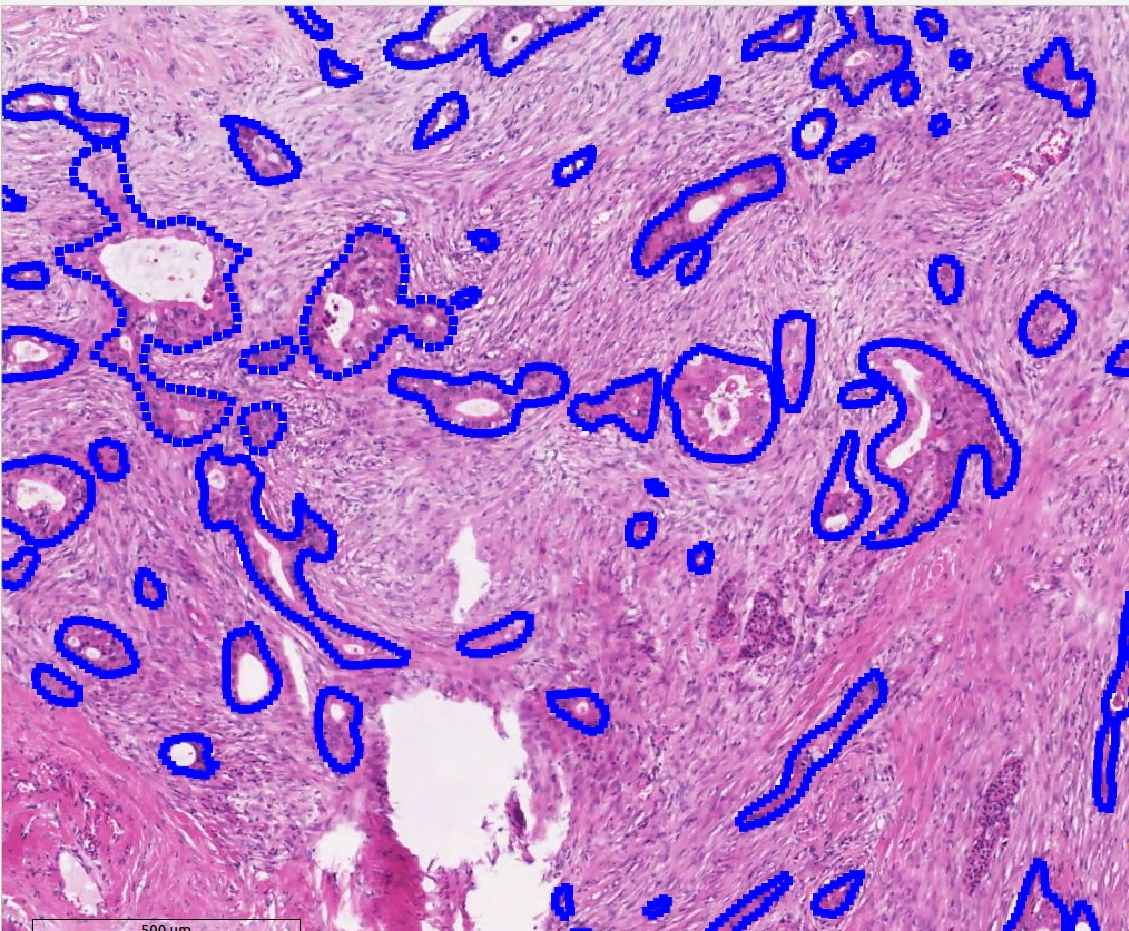


Supplementary Figure 2. **The diagram of WSIs annotation**

Pathologists used irregular curves to encircle the cancerous tissue, and image cutting was performed on these annotated regions to get patches.

**
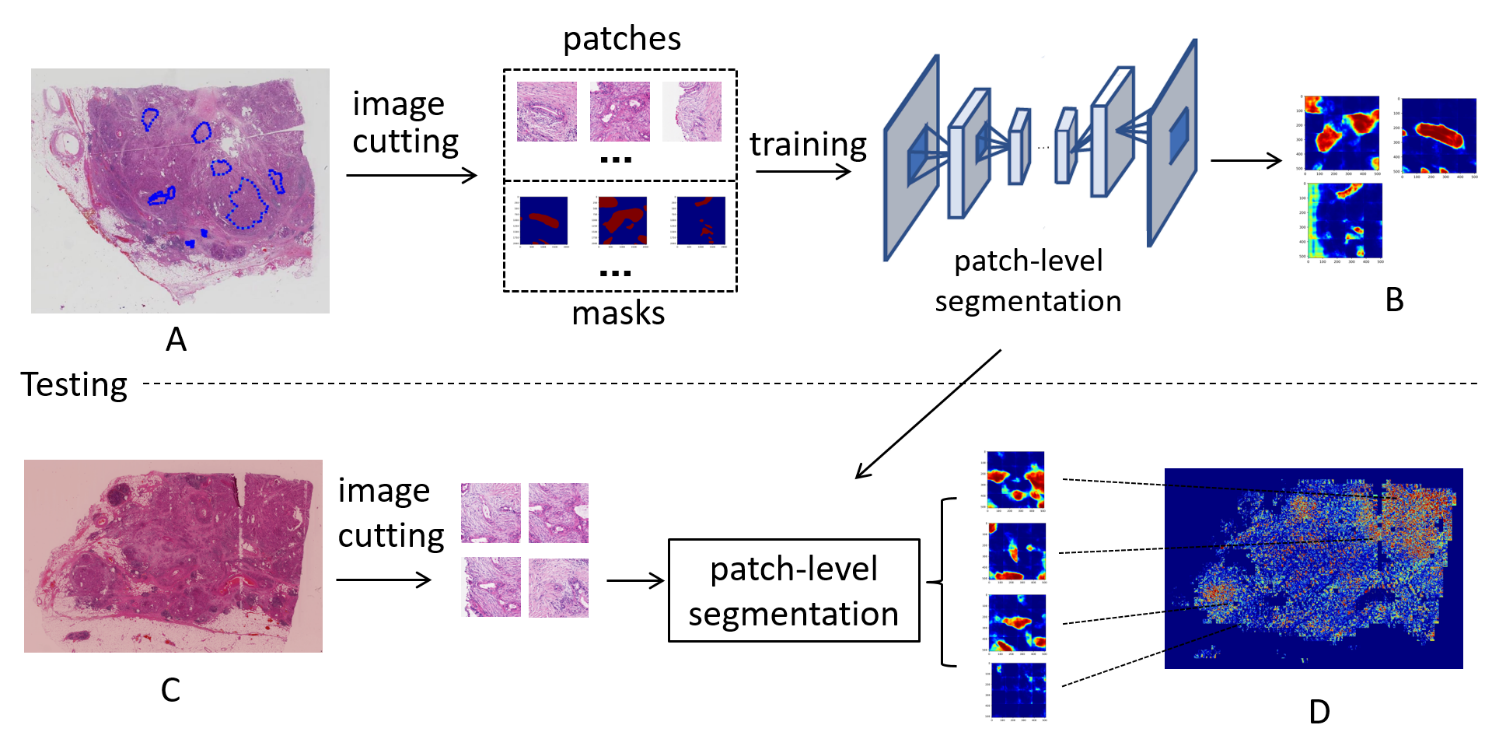
**

Supplementary Figure 3. **The framework of segmentation task**

(**A**) A pathologist’s annotation for a WSI. (**B**) Heatmap of the patch predicted by our method. (**C**) Raw test WSI. (**D**) Heatmap of the WSI.

For patch-level segmentation, we chose U-Net as the patch-level segmentation model. Then we obtained the heatmap of each patch predicted by our segmentation method. For WSI-level segmentation, we just merged the patch-level segmentation results of all patches.
